# Developing Embodied Conversational Agents for Coaching People in a Healthy Lifestyle: Scoping Review

**DOI:** 10.2196/14058

**Published:** 2020-02-05

**Authors:** Lean L Kramer, Silke ter Stal, Bob C Mulder, Emely de Vet, Lex van Velsen

**Affiliations:** 1 Consumption and Healthy Lifestyles Wageningen University & Research Wageningen Netherlands; 2 Strategic Communication Wageningen University & Research Wageningen Netherlands; 3 eHealth Cluster Roessingh Research and Development Enschede Netherlands; 4 Faculty of Electrical Engineering Mathematics and Computer Science University of Twente Enschede Netherlands

**Keywords:** embodied conversational agent, virtual agent, lifestyle, health behavior, eHealth, chatbots

## Abstract

**Background:**

Embodied conversational agents (ECAs) are animated computer characters that simulate face-to-face counseling. Owing to their capacity to establish and maintain an empathic relationship, they are deemed to be a promising tool for starting and maintaining a healthy lifestyle.

**Objective:**

This review aimed to identify the current practices in designing and evaluating ECAs for coaching people in a healthy lifestyle and provide an overview of their efficacy (on behavioral, knowledge, and motivational parameters) and use (on usability, usage, and user satisfaction parameters).

**Methods:**

We used the Arksey and O’Malley framework to conduct a scoping review. PsycINFO, Medical Literature Analysis and Retrieval System Online, and Scopus were searched with a combination of terms related to ECA and lifestyle. Initially, 1789 unique studies were identified; 20 studies were included.

**Results:**

Most often, ECAs targeted physical activity (n=16) and had the appearance of a middle-aged African American woman (n=13). Multiple behavior change techniques (median=3) and theories or principles (median=3) were applied, but their interpretation and application were usually not reported. ECAs seemed to be designed for the end user rather than with the end user. Stakeholders were usually not involved. A total of 7 out of 15 studies reported better efficacy outcomes for the intervention group, and 5 out of 8 studies reported better use-related outcomes, as compared with the control group.

**Conclusions:**

ECAs are a promising tool for persuasive communication in the health domain. This review provided valuable insights into the current developmental processes, and it recommends the use of human-centered, stakeholder-inclusive design approaches, along with reporting on the design activities in a systematic and comprehensive manner. The gaps in knowledge were identified on the working mechanisms of intervention components and the right timing and frequency of coaching.

## Introduction

### Background

**Figure 1:**
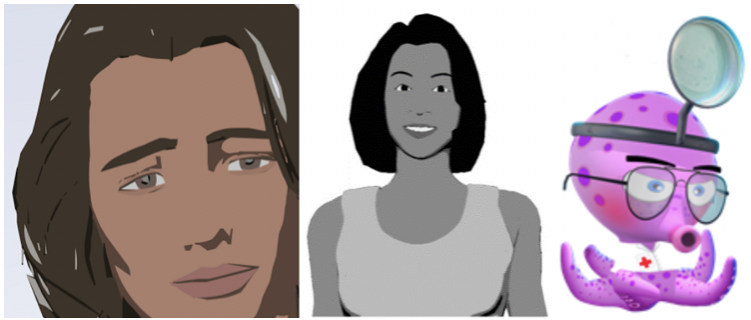
Example of embodied conversational agents.

Public health would substantially improve if a large number of people adopted a healthy lifestyle, encompassing among others, ample physical activity, and healthy diets [1]. To initiate or coach such change, embodied conversational agents (ECAs) can be a valuable tool. ECAs can be defined as “more or less autonomous and intelligent software entities with an embodiment used to communicate with the user” [2]. Examples include those given in Figure 1; From left to right: *Laura* [3], *Gabby* [4], and an anonymous octopus [5]. An example of an early ECA is *Laura* [3]. Laura interacts daily with users to motivate them to be more physically active. She uses several relational behaviors, such as social dialogue, feedback, humor, facial expressions, and body language. Through these behaviors, users establish and maintain a meaningful relationship [3]. What makes ECAs unique for coaching people with respect to their health is this capacity of establishing and maintaining an empathic relationship [3], a relationship characteristic proven to be the most crucial factor for successful lifestyle coaching [6]. In addition, ECAs are available 24×7. Consequently, they can offer empathic support when it matters most: immediately before or after specific behavior, which maximizes impact [7].

Despite the promising role ECAs can play in coaching people for a healthy lifestyle, literature that discusses how to develop them and demonstrates their effectiveness is scarce. A review by Provoost et al [8] provides some insight into the developmental processes and evidence base of ECAs for coaching people with mental disorders. They suggest that the more rigorous studies put little emphasis on design and that evidence on clinical effectiveness remained sparse [8]. In the educational context, Johnson and Lester [9] state that there is a significant body of experience and research findings related to pedagogical agents. However, similar to the health context, many questions remain about when pedagogical agents are most effective and how they should be designed and used to maximize effectiveness. Literature on development and effectiveness is essential to create ECAs that can have a high level of impact and uptake, a problem with which electronic health (eHealth) interventions constantly struggle [10]. The cause for this low impact and uptake is often attributed to a misfit among technological, human, and contextual factors during development [11,12]. Different authors have therefore recommended to apply a human-centered and stakeholder-inclusive design approach, as well as to incorporate persuasive design features in the technology [11,13,14].

### Objectives

This scoping review identifies the current developmental practices of ECAs for coaching people in a healthy lifestyle, and it provides an overview of their efficacy and use-related outcomes. For researchers, this review provides an overview of the potential ECAs have to change people’s lifestyle and identifies the most urgent research questions related to this domain. For practitioners, the review will lead to actionable advice for devising a development trajectory for this type of ECAs.

## Methods

### Study Design

The Arksey and O’Malley framework for scoping reviews [15] was adopted, which distinguishes 5 different stages: (1) identifying the research question, (2) identifying relevant studies, (3) selecting studies, (4) charting the data, and (5) collating, summarizing, and reporting the results.

#### Identifying the Research Question

The research question was identified from a preliminary scan of the literature, which showed a lack of insight into and description of best practices regarding the current development processes. The question that will be answered is as follows: How are ECAs for coaching people in a healthy lifestyle designed and evaluated?

#### Identifying Relevant Studies

To identify relevant studies, a data logbook was created, comprising specific instructions, a plan, a term list, and a data-charting form. The databases used to locate the relevant literature were as follows: PsycINFO, because of its comprehensive library of psychological science; Medical Literature Analysis and Retrieval System Online, because of its wide coverage of scientific journals in the health domain; and Scopus, because of its multidisciplinary scope. The databases were searched for peer-reviewed journal articles written in English, with a combination of terms related to *ECA* and *lifestyle*. The keywords were identified based on a preliminary literature scan and in consultation with a research librarian to obtain a comprehensive list of potential sources (see Multimedia Appendix 1). In addition, we applied the snowball method.

### Study Selection

Inclusion criteria were implemented by selecting different options and limits during the search (see Multimedia Appendix 1). The results of the search query were uploaded into the EndNote reference manager (Thomson Reuters) and independently assessed by 2 reviewers (LK and SS) to decide on their inclusion based on title, abstract, and full text. Conflicts between the 2 reviewers were identified after each step, independently; arguments were formulated per study and then discussed and resolved. This process was documented in the logbook. To find relevant studies that describe an intervention with an ECA in the healthy lifestyle domain, the following exclusion criteria were applied: (1) there is no report on primary data, (2) there is no intervention, (3) the intervention does not include an ECA (a “more or less autonomous and intelligent software entities with an embodiment used to communicate with the user”) [2], and (4) the ECA is not used in a lifestyle health behavior context (eg, tobacco use, physical (in)activity, alcohol consumption, and diet) [4].

#### Charting the Data and Collating and Summarizing the Results

Data from the selected studies were charted independently by 2 reviewers (LK and BM). The following categories were a part of the data-charting form: (1) article information, (2) study information, (3) general description of an ECA, (4) information regarding the visual design and content, (5) support offered by the ECA, (6) information procedures to introduce the ECA to its user, and (7) formative evaluation. Each category could be completed by selecting the applicable predefined content, based on the study by Provoost et al [8] (see Multimedia Appendix 2 for all options). Conflicts between reviewers were identified and resolved by jointly reviewing the component and discussing the conflict, and these were documented in the logbook. When all the studies had been inventoried, we analyzed them thematically, which resulted in 3 topics. The first topic describes the different definitions and descriptions that were used for ECAs. The second topic describes the design and design processes of the ECAs, including their embodiment and communication modalities, applied theories, principles, and behavior change techniques (BCTs). To create a uniform language among the BCTs, the BCT Taxonomy (v1) from Michie et al [16] was used. The third topic describes the procedures, evaluation processes, and the efficacy and use-related outcomes.

## Results

### Study Selection and Characteristics

**Figure 2:**
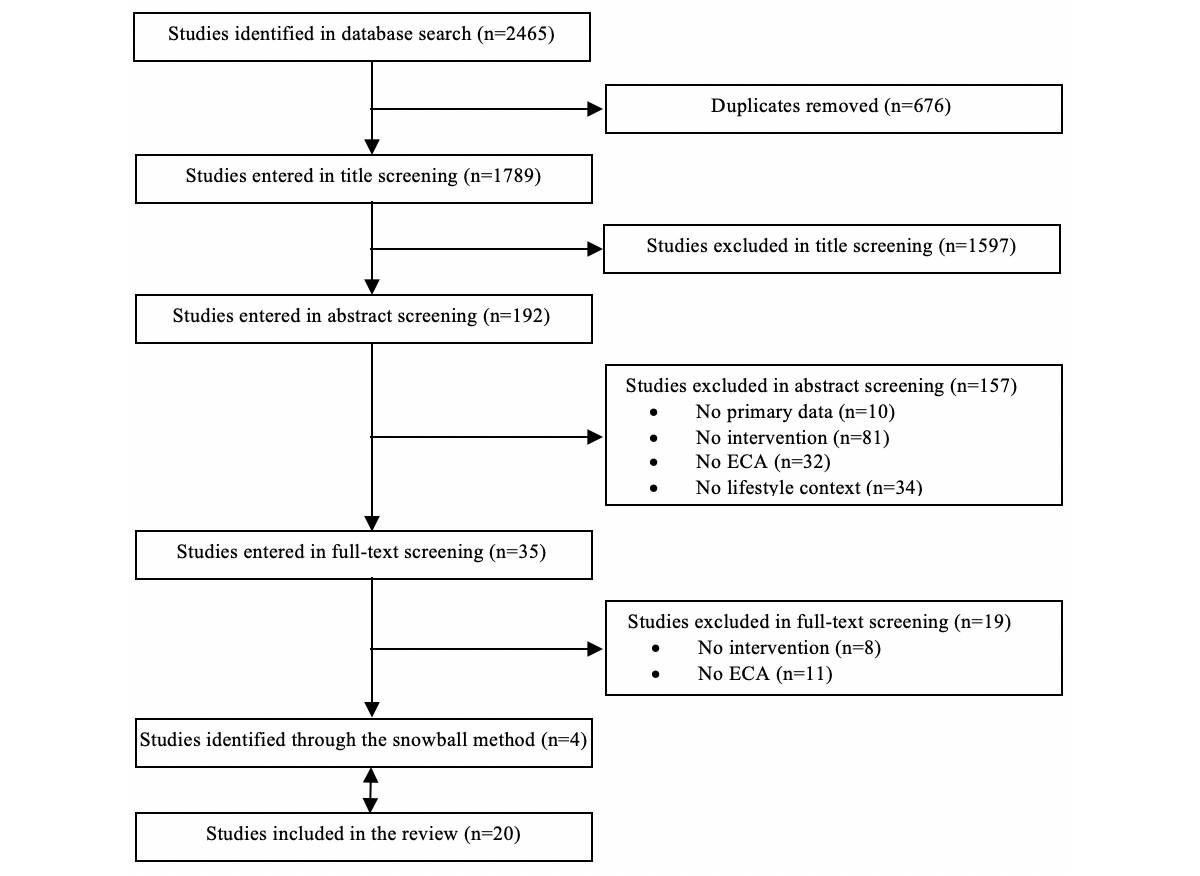
Flowchart describing study screening and selection.

Figure 2 charts the screening and selection process. In total, 1789 unique studies were identified in the database search. Title and abstract screening resulted in the exclusion of 1754 studies. The remaining 35 studies were screened in full. Of those, 19 studies were excluded as the studies were not an intervention or did not include an ECA. This resulted in a total of 16 studies. One of these studies [4] described both a rehospitalization and a physical activity trial. As the first is not a lifestyle behavior, only the second trial was included in the analysis. A total of 4 more studies were found through snowballing [17-20]. This resulted in a total of 20 studies that were included in this review (see Multimedia Appendix 3 for a complete overview of the study characteristics).

The first studies were published in 2005 [3,17,21]. All the studies were either performed in the United States [3,4,17-19,21-31] or in the Netherlands [5,20,32,33]. Of all the studies performed in the United States, except for 1 study [26], TW Bickmore was listed as the author. A total of 13 studies were in the pilot phase [3,4,17-19,21,24-26,28,30-32], 1 study was in the development phase [22], and 6 studies were in in the evaluation phase [20,23,27,29,31,32]. Thus, none of the studies described the implementation or had actually implemented their ECA in practice. One ECA was used in a community setting and could be accessed via a computer kiosk [29]. All other ECAs were used at home and could be accessed via a website [20,24,26,28,30-32], or software installed on a PC [3,17,19,21-23,25], tablet [4,18,27], or mobile phone [33]. Only 1 ECA was part of an overarching platform, accessible via a website and an Android app [5]. Most studies targeted physical activity [3-5,17-23,25,27,29-33]. Other lifestyle behaviors were nutrition [5,20,25,30], mindfulness [26,30], preconception care [24,28], stress [30], blood glucose monitoring [5], and sun protection [31]. Moreover, one specific study targeted healthy lifestyles among diabetes patients. Patients may differ in their needs for lifestyle support compared with healthy individuals. The diversity in focus and target groups limits the comparability among the studies, and future research could help expand the evidence base for specific ECAs. Study designs varied from a randomized controlled trial (RCT) [3,4,17,19-23,25-30,32,33] to a pretest-posttest design, either with [31] or without control a control group [5,18,30]. Sample size ranged from 9 to 958 participants (median=60.5). Study duration lasted from 4 weeks to 36 months (median=8 weeks).

### Descriptions and Definitions

Across the studies, 9 different names were used to describe an ECA, although the definitions were rather similar. A total of 6 studies used the name *embodied conversational agent* [3,4,19,26,27,30], whereas the other studies used different names: *relational agent* [3,17,21,22,31], *virtual coach* [5,23], *virtual exercise coach* [18], *virtual avatar* [32], *virtual patient advocate* [24], *conversational agent* [28], *animated conversational agent* [25], *virtual advisor* [29], *personal digital coach* [33], and *persuasive computer assistant* [20]. A total of 6 studies did not provide a definition for an ECA [5,17,20,23,25,32]. All other studies referred to earlier with TW Bickmore listed as the author used variations of “an interactive, animated computer character that simulates face-to-face counseling” [5].

### Design and Design Processes

#### Design: Embodiment, Communication Modality, Content, and Communication Strategy

All studies provided a screenshot of the agent. These images show that the embodiments of all ECAs were rather similar; 13 ECAs had the appearance of a middle-aged African American woman: 3 agents had an appearance similar to *Laura* [3,17,21], 6 agents were similar to *Gabby* [4,18,24,27,28,30], and 5 agents were similar to *Carmen* [19,22,23,25,29]. Other ECAs were a white woman [26,32,33], a cat (the virtual iCat) [20], and an octopus [5]. In addition, 1 study used 4 different ECAs, using race and gender to match participants to one of the agents [31]. Thus, in total, there were 9 different agents. These agents communicated through text [5,19,20,32] or speech [3,24,31], or they allowed the user to choose between text or speech [33]. For the iCat, no information was provided [20]. Regarding the communication modalities, all but 1 agent [5] used facial and gaze expressions; in addition, only a few used hand and body gestures [3,31]. Most users communicated with the agent by choosing a single response from a fixed list of responses [3,19,24,26,32]. Some agents also offered the possibility to type an answer in a textbox [26,32]. A total of 2 studies did not provide any information on how users could communicate with the agent [20,31].

Behavioral theories or therapy-derived principles were applied in a majority of the ECAs to drive their content and communication strategy. In total, 17 different theories and principles were mentioned in the 20 studies (median=3, range 1-4; see Multimedia Appendix 3 for an overview). A total of 3 studies did not mention any theory or principle [4,22,27], whereas the remaining studies did not discuss their interpretation or application. It is therefore unclear what role theories play in the design process. The Transtheoretical Model was mentioned most often [17,19,24,25,28,29,31,33]; its application was, for example, described as “educational information based on current progress” [19]. Other theories or principles used more than once were as follows: Motivational Interviewing [20,25,28,30,32], for example, “cooperative feedback on the diary entries following the motivational interviewing concept” [20]; Social Cognitive Theory [19,23,25,29] and Behavioral Theory [17,23], for example, “the script employs behavioral and social cognitive strategies demonstrated in the literature to promote exercise behavior change” [23]; and Cognitive Behavioral Therapy [17,18], for example, “the agent (…) uses a number of additional cognitive-behavioral techniques for health behavior change” [17]. In addition to or based on the theories and principles, the content and communication strategy also comprised BCTs. In total, 24 different BCTs were mentioned in the 20 studies (median=3, range 2-10; see Multimedia Appendix 3 for an overview). Again, 3 studies did not report any techniques [3,21,22]; the remaining studies did so very briefly. Furthermore, no uniform language was used to describe BCTs; therefore, it remained unclear how the BCTs were operationalized. *Goal setting* was mentioned most often [4,5,17-20,23,25,27-32], and it was, for example, described as “weekly goals for exercise” [31]. Other frequently used BCTs were *information about health consequences* [5,17-20,23-26,28,30,32], for example, “educational content about physical activity” [17]; *problem solving* [17,18,23,25-28,30-32], for example, “tailored strategies that addressed related barriers” [31]; *social reward* [5,17,19,20,23,26,27,29,31], for example, “positive reinforcement” [23]; *feedback on behavior* [4,5,18-20,29,31,33], for example, “feedback about the behavior of the users” [33]; *social support (practical)* [5,18,27,28,30,31,33], for example, “exercise tip of the day” [18]; and *self-monitoring of behavior* [5,17,20,29,31,33], for example, “self-monitoring charts” [27].

#### Design Processes

Regarding the design processes of the embodiment and communication modalities of the 9 different ECAs, 5 studies did not provide any information [19,20,26,31,33]. There was 1 study that provided some information, although very briefly: “The design of the gamification and coaching platform adheres to basic principles of healthcare, design principles for serious gaming as well as design principles for behavior change support systems” [5]. The remaining 3 studies did provide detailed information. A total of 2 studies reported on the design and the results of a focus group with end users, which resulted in the current appearance of the agent [24,32]. The third study reported on the findings of various design methods: “Studies of interactions between human exercise trainers and their clients,” a survey with end users and a literature review [3].

Regarding the design process of the content and communication strategies of the 20 ECAs, 9 studies did not provide any information [4,5,18,19,25-27,29,31]. In all, 2 studies [22,28] referred to other publications [17,24], which were also included in this review. Two studies each referred to a study, which is not part of this review, in which the design process is described: The first study [32] refers to a publication describing a pilot study on autonomous motivation and appreciation [34], and the second study [32] refers to a publication describing a survey with end users on the situation and timing of feedback [35]. A total of 3 studies provided some, very brief, information: “The ECA system for this study was adapted from the Gabby Preconception Health Care system’s dialogue scripts and media” [30]; “Both the personal lifestyle goals and the feedback were evaluated and improved where necessary by a dietician” [20]; and “The 60 pages of educational content were assembled from publicly available web pages on exercise topics (...)” [3]. A total of 3 similar studies provided only some brief information, but these did include an interdisciplinary collaboration involving physicians, computer scientists, and exercise trainers to ensure adherence to best practices [17,21,23]. A final study used multiple methods and provided detailed information. It describes how they used scripts and media tools from previous studies and reports on a focus group in which they tested the content with end users [24].

### Evaluation Processes and Outcomes

#### Evaluation Processes: Procedures and Measurement

A total of 7 studies did not provide any information regarding the procedures that were undertaken to introduce the ECA to its user [20,21,23,26,28,31,32]. The remaining studies only provided a short description. Most of the studies that did provide some information described a demonstration on how to use the system, which took place at the start of the study [3-5,17-19,22,25,27,29,30], for example, “participants were instructed on how to use the ECA system” [23]. For 1 study, participants were given “a brief group demonstration” [24]. However, another study sent “a user manual about the installation of the software” via email [33]. Another study sent instructions via email after 3 days of use [20]. Only 2 studies reported on assisting the user with user problems during the study: 1 study described contacting the user when the user stopped using the ECA [23]; the other study involved set times to check for technical issues [18].

Contrary to the procedures, the measurement of efficacy (behavioral, knowledge, and motivational parameters) and use (usability, usage, and user satisfaction parameters) was well described in all the studies (see Multimedia Appendix 2 for concept definitions, Multimedia Appendix 3 for an overview of all parameters, and Table 1 for a summary).

All the studies assessed a combination of multiple parameters (median=4.5, range 2-6). One study [29] only described a protocol [19]; therefore, it was not considered in this section.

Regarding the efficacy parameters, behavior was assessed in all but 5 studies [4,5,24,26,31]. An example is the number of steps assessed by either a pedometer [3,17,21-23,25,27] or activity monitor [33]. Behavior was also assessed by self-report, usually in a questionnaire format [17,19,21,23,25,28,32], for example, “the usual weekly minutes of walking over the previous 4 weeks” [19]. Furthermore, a walking test for both distance and speed was used in 1 study [18]. Knowledge of the participant was assessed in 3 studies [20,26,30], and it was operationalized as lifestyle knowledge [20], food knowledge [30], or “conceptual and practical knowledge about mindfulness meditation” [26]. Knowledge was assessed by either a questionnaire [20,26] or an interview [30]. There were 4 studies describing users’ motivation to change [19,20,24,26], including *stage of change* [24,26], *motivation to fill in diary* [20], and *motivation processes of change* [19], which were all assessed by a questionnaire.

**Table 1:** Differences in total number of efficacy and use-related outcomes between intervention and control group.

Outcome variable and measure		Significant^a^	Nonsignificant^b^	No data^c^
**Behavior**				
	Interview	—^d^	1	—
	Other	—	1	1
	Pedometer	2	3	2
	Questionnaire	3	—	—
	Self-report	—	1	—
**Knowledge**				
	Interview	—	1	—
	Questionnaire	—	1	1
**Motivation**				
	Questionnaire	2	—	2
**Usability**				
	Not reported	—	—	1
	Questionnaire	1	—	4
**Usage**				
	Log files	4	1	11
**User satisfaction**				
	Interview	—	—	2
	Questionnaire	—	2	14

^a^Significant positive difference between intervention group with and control group without an embodied conversational agent.

^b^Nonsignificant difference between intervention group with and control group without an embodied conversational agent.

^c^Difference not applicable or not reported.

^d^An absence of outcome measure for the outcome variable.

Regarding the use-related parameters, 6 studies assessed whether users had had trouble using the intervention [3,19,20,24,25,33] because of technical issues or lack of technical knowledge. Usability was assessed by a questionnaire [3,20,24,25,33]. One study did not report on how it assessed usability [19]. Usage was assessed in all but 3 studies [25,31,32]. All the studies assessed how and how often the intervention was used by log files. User satisfaction was assessed in all but 1 study [20]. Most often, single items were used to assess users’ satisfaction with the interventions [3,4,17-19,21-28,30,32,33]. User satisfaction concerns items related to constructs such as liking, trust, and desire to continue using the ECA, for example, “How much do you trust Gabby?” [24]. Other methods used were interviews [3,5,17,25,30,31,33] and a focus group with end users [5].

#### Evaluation Outcomes: Efficacy and Use Related

When comparing the intervention group with an ECA with a control group without an ECA, more significant positive (n=12) than nonsignificant effects were found (n=11; see Table 1). In other words, in 12 studies, the intervention groups showed improvement compared with the control group, whereas in 11 studies, there were no differences. However, for a majority of the outcome measures, this comparison was either not applicable as there was no control group without an ECA (n=37) or the significance level was not reported (n=4). Overall, 7 out of 15 studies reported better efficacy outcomes for the intervention group, and 5 out of 8 studies reported better use-related outcomes, compared with the control group.

Regarding the outcomes on behavior, it was found that participants using an ECA identified more preconception risks [28] compared with control participants only receiving an email. Both the studies on nutrition found no differences in eating patterns [30] and adherence to diet [20] between participants who had engaged with the ECA and participants who had not. In physical activity–related studies, 4 [19,23,27,32] out of 8 studies [3,17,19,21,23,27,32,33] found a positive difference in physical activity levels between participants who had engaged with the ECA and participants who had not. Regarding outcomes on knowledge, participants in the intervention arm did not score higher on lifestyle literacy, compared with control participants who had the same intervention without an ECA providing feedback [20]. Similarly, the food literacy outcomes of the participants in the intervention arm were not higher than those of the participants in the control arm, who had reviewed the same content with a research assistant once and received a CD with similar meditation recordings [30]. For motivational outcomes, the motivation to fill in a diary [20] and use of motivational behavior change strategies were higher for participants in the intervention arm [19] than for participants in the control arm.

Regarding the use-related outcomes, it was found that participants with an ECA considered the intervention as easier to use [20], compared with control participants who had the same intervention without an ECA providing feedback. Participants with an ECA also used the intervention more frequently [17,20,21,26]. However, 1 study showed the opposite and reported a nonsignificant effect for uptake on impact [23]. A total of 6 studies measured the usage over time, all showing a decrease [3,4,19,22,23,27], for example, “A typical usage pattern was daily during the first week, tapering off to once or twice a week by the end of the study period” [3]. A total of 4 studies reported the average duration of a session, ranging from 12 min [24,29] to 19 min [26,28]. The average number of sessions during the intervention period was mentioned in 6 studies [18,19,23,24,27,28], which was a median of 27.5 sessions (range 8-36). The intervention period of these studies was a median of 8.6 weeks (range 4 weeks-4 months), and this was unrelated to the number of sessions. Participants interacting with an ECA did not report higher satisfaction outcomes [23], compared with control participants who could also view graphs and set goals without interacting with an ECA. In addition, participants in the intervention arm were equally satisfied with the ECA for improving health behaviors [30].

## Discussion

### Principal Findings

This scoping review charted the design and evaluation field of ECAs for coaching people in a healthy lifestyle. In total, 20 relevant studies were identified and analyzed. One could argue that the lack of diversity in research teams limits the external validity of the scoping review. However, although the work in this field is dominated by 1 research group, a careful comparison between research groups showed no differences in design and evaluation processes, as well as in outcomes (see Multimedia Appendix 3). We therefore conclude that the developmental processes described in this review are a realistic reflection of the field. Regarding the design, we found that studies often applied multiple theories or principles, but they did not report on their interpretation and application. Human-centered and stakeholder-inclusive design approaches tended to be unused. Regarding the evaluation, a combination of efficacy and use-related outcomes was assessed, usually in an RCT. However, rather than evaluating specific components, the intervention was evaluated as a whole. Overall, the studies included suggest that ECAs for coaching people in a healthy lifestyle can make an intervention more engaging, although evidence on their effectiveness remains inconclusive.

Myriad theories and therapy-derived principles were applied for creating ECAs’ content and communication strategy. As it is difficult to determine what theory or principle best fits a specific context and as it is reasonable to assume that different contexts require the use of different theories and principles, we do not consider this diversity a problematic issue. However, what we do see as problematic is the lack of detail with which the incorporation of these theories and principles into functional or content design of an ECA is reported. If how exactly an ECA works remains unclear, it will be difficult to learn from others’ efforts or interpret the outcomes of evaluations performed with an ECA. This prevents knowledge accumulation about ECAs in general, as well as specific knowledge accumulation about which theories and principles are most appropriate in which contexts. A similar conclusion can be drawn with respect to the design process of ECAs. The design of an ECA can have a major effect on both impact and uptake. On the basis of empirical results of different studies on the appearance of ECAs, Baylor concludes that different appearances lead to different outcomes in terms of motivation and behavior change [36]. Unfortunately, reporting on the design activities and their results is generally incomplete or missing, thereby limiting the options for replication and learning from others’ work. It is therefore recommended that future ECA work should not only present results on the efficacy of the ECA but also on the process leading to the design and content of the ECA.

With respect to the evaluation of ECAs for coaching people in a healthy lifestyle, we made a distinction between the results in ECAs’ efficacy and use-related parameters. ECA outcome efficacy shows a nonconclusive picture, operationalized as, for example, physical activity measured by an activity monitor, knowledge about mindfulness meditation as assessed via a survey, or diabetes-related emotional distress. About half of the evaluation outcomes show a significantly positive result for using an ECA, whereas the other half of the outcomes do not provide positive evidence. With regard to use-related outcomes, the evaluations do show a positive picture, where the majority of the studies indicate that the use of an ECA leads to higher ratings of usability or a higher degree of use. With regard to the efficacy-related outcomes, motivation to change had successfully improved in a majority of the studies, whereas health behavior and health literacy had not. On the basis of the existent evaluations, we can therefore state that ECAs do not necessarily lead to improved health outcomes; however, the intervention will at least be more engaging. This is in accordance with Provoost et al, based on their review of ECAs in clinical psychology and their evidence base [8].

### Beyond the State of the Art

We found that end users are normally not involved with the visual design and content of the ECA. Rather, the ECAs were designed by professionals behind a desk. This practice contradicts human-centered or collaborative design approaches that are assumed to lead to technology appealing to and fitting the perspectives of the end users [37]. This consequently maximizes the chance of successful uptake of the technology [10]. In the literature, several practical approaches for human-centered design for eHealth are provided, such as the Centre for eHealth and Wellbeing roadmap [11] or Integrate, Design, Assess, and Share [38], as well as a rich collection of case studies in which these approaches have been used [39,40]. The field of developing and evaluating ECAs for eHealth would highly benefit from the reporting of similar case studies in diverse contexts.

We found that the evidence for using ECAs for coaching people in a healthy lifestyle remains inconclusive and that it is unclear which (combination of) components caused a (lack of) behavior change. However, this problem is neither new nor exclusive to the field of ECAs; this so-called *black box* phenomenon has been acknowledged for eHealth interventions in general [32,41]. Rather than evaluating an eHealth technology or ECAs for health purposes as a whole, an evaluation should focus on gaining insight into the effectiveness of the technology’s or ECA’s main or constituent components. A more fine-grained evaluation can be achieved by means of a factorial design, as this allows researchers to deliver specific intervention components to different groups of users [42]. Another strategy is to collect log files on usage time and patterns to identify the technology components that affect (non)use [37].

The studies in our review suggest that ECAs can make an eHealth intervention, aimed at improving people’s lifestyle, more engaging. This is possibly because of the capacity of ECAs to establish and maintain an empathic relationship [3]. However, one can wonder how lasting this engagement is. Providing an ECA may have a novelty effect; thus, the engaging effect may wear off over time, resulting in decreased adherence, which is common for eHealth interventions [10]. Studying the use, effectiveness, and user experience of working with an ECA for coaching people in a healthy lifestyle for a prolonged period and in a realistic setting would provide inputs for answering these questions. Both researchers and eHealth developers need to find these answers to identify the persuasive goals that ECAs can serve best and to know how such ECAs should be developed to create engagement and a lasting effect.

### Recommendations for Future Design and Research

On the basis of the findings of this review, we formulate several recommendations for future design and research. With respect to the development of ECAs for coaching people in a healthy lifestyle, we recommend the use of human-centered, stakeholder-inclusive design approaches, as well as reporting on the design activities in a systematic and comprehensive manner. This will allow others to learn from previous efforts. With respect to evaluation, there is a need to open the *black box* that is now pervasive among studies that delve into the efficacy of ECAs in improving health-related lifestyle. This means that evaluation reports need to specify which features are considered the main components of the eHealth intervention with an ECA and what theoretical foundation lies beneath these features, the ECA, and its persuasive tactic. Thereafter, during the data analysis phase of an evaluation, these features should be linked to measures of efficacy, use, and the user experience, to grasp whether the ECA works and why (not). Only in this way, a single evaluation can become valuable, both within and beyond its specific context.

Besides these general recommendations, we have also identified several specific research questions. As we mentioned in the introduction, the 24×7 availability of an ECA and its potential to deliver coaching at exactly the right moment (ie, just before or after specific behavior) make it a potentially valuable addition to the persuasive tool kit that eHealth developers have at hand. However, none of the included studies focused on identifying the exact right timing for a specific type of content. Should we always try to prevent negative behavior, thereby running the risk that the ECA may become annoying? Should we always acknowledge positive behavior, thereby running the risk that the ECA loses credibility? Finding the answers to these questions related to timing and frequency of use will allow us to create persuasive tactics for ECAs, which are in line with the tolerance levels and needs of end users. Furthermore, to fully understand the novelty effect that the introduction of an ECA may bring and to grasp the development of behavior change over time, longitudinal studies need to be performed. Ideally, these studies are (partly) in depth and qualitative to generate hypotheses for a novel field that can then be confirmed in large-scale quantitative studies afterward.

### Limitations

The first limitation is that we might have missed relevant studies. The applied search strategy might have influenced our findings, as it is plausible that ongoing studies are only published in conference proceedings. The applied search string might also have influenced our findings. During the stage of identifying relevant keywords, we already found a variety of terms used to describe (comparable) ECAs. With the help of a librarian, we therefore tried to mitigate this risk by setting up a comprehensive list based on an initial search. In the end, we identified 9 different terms in the studies included, although the definitions were rather similar. As a recommendation for future work, we propose to use the term *ECAs* as the uniform term for “more or less autonomous and intelligent software entities with an embodiment used to communicate with the user” [2].

The second limitation relates to the identification of BCTs. They were rather difficult to identify as they were often mentioned summarily in the text or within images, and no uniform language was used, for example, we could only code *Tailored strategies that addressed related barriers* [31] as *problem solving*, according to the BCT Taxonomy (v1) from the study by Michie et al [16]. Further descriptions were usually not provided.

### Conclusions

ECAs are a promising tool for persuasive communication in the health domain. This scoping review provided valuable insight into the current development processes and evaluation outcomes. On the basis of these results, we offer multiple recommendations for future research agendas. We hope that the lessons from this review will further shape the novel field of using ECAs within the eHealth context.

## Appendix

Multimedia Appendix 1 Search string and database search.

Multimedia Appendix 2 Term list data-charting form.

Multimedia Appendix 3 Overview of studies.

## Abbreviations

BCT: behavior change technique
ECA: embodied conversational agent
eHealth: electronic health
RCT: randomized controlled trial
ZonMw: The Netherlands Association for Health Research and Development
