# Supplementary material for: Developing Embodied Conversational Agents for Coaching People in a Healthy Lifestyle: Scoping Review
Source: J Med Internet Res. 2020 Feb 5;22(2):e14058. doi: 10.2196/14058 (PMC7055763; doi:10.2196/14058)
Supplement: Multimedia Appendix 1 [file jmir_v22i2e14058_app1.docx]

## Multimedia Appendix 1. Search string and database search

### Search string

(“animated character*” OR “artificial agent*” OR “artificial intelligence assistant*” OR “assistant chat program*” OR “assistive social agent*” OR “chatbot program*” OR  “communicative agent*” OR “companion agent” OR  “companion assistant*” OR “conversational agent*” OR  “conversational assistant*” OR  “digital assistant*” OR “embodied agent*” OR “embodied conversational agent*” OR “interactive agent*” OR “interface agent*” OR “online chat program*” OR “pedagogical agent*” OR “persuasive ECA” OR “relational agent*” OR “relational assistant*” OR “software agent*” OR “virtual agent*” OR “virtual assistant*” OR  “virtual character” OR “virtual coach*” OR “virtual counselor*” OR  “virtual health counselor*” OR “virtual health agent*” OR  “virtual health coach” OR “virtual human” OR “virtual patient advocate*” OR “virtual therapist*” OR “virtual web assistant*” AND “activ*” OR “alcohol” OR “behavio?r change” OR “diet” OR “exercise*” OR "health*” OR “lifestyle” OR “mindful*” OR “nutrition” OR “obese” OR “obesity” OR “overweight” OR “pedestrian" OR "physical activity" OR “sedentar*” OR “sleep” OR “smok*” OR “sport*” OR “walk*” OR “weight loss”)

### Options and limits per database

**Table 1.** Options and limits selected per database.

| Database | Field | Options and limits |
| --- | --- | --- |
| PsycINFO |  |  |
|  | Search in | Title or abstract (ECA) and abstract (lifestyle) |
|  | Language | English |
|  | Document type | Journal article |
|  |  |  |
| MEDLINE |  |  |
|  | Search in | Title (ECA) and abstract (lifestyle) |
|  | Language | English |
|  | Document type | Journal article |
|  |  |  |
| Scopus |  |  |
|  | Search in | Title and abstract |
|  | Language | English |
|  | Document type | Article |
