# Supplementary material for: Developing Embodied Conversational Agents for Coaching People in a Healthy Lifestyle: Scoping Review
Source: J Med Internet Res. 2020 Feb 5;22(2):e14058. doi: 10.2196/14058 (PMC7055763; doi:10.2196/14058)
Supplement: Multimedia Appendix 2 [file jmir_v22i2e14058_app2.docx]

## Multimedia Appendix 2. Term list data-charting form

**Table 1.** Categories, components and definitions used for data charting.

| Category and component | Definition |
| --- | --- |
| **Article information** |  |
| Publication information | The APA reference of the article |
| Country | The country in which the study took place |
| Aim | What the study aimed to find out |
|  |  |
| **Study information** |  |
| Duration | The number of weeks or months the study lasted per study part |
| Setting | The setting in which the ECA was tested |
| Recruitment | The recruitment strategy used to recruit participants |
| Race | The social demographic background of the majority of the participants |
| Gender | The gender of the participants included in the allocation |
| Number | The number of participants in the intervention and follow-up, included in the allocation |
| Age | The age category of the participants:  - Preschoolers: 0 - 4 years  - Children: 5 - 12 years  - Adolescents: 13 - 17 years  - Young adults: 18 - 24 years  - Adults: 25 - 64 years  - Elderly: >65 years |
| Study design | Method used to evaluate the intervention |
|  |  |
|  |  |
| **Description ECA** |  |
| Term ECA | The term which is used to name an ECA in general |
| Description ECA | The description which is used to describe an ECA in general |
|  |  |
|  |  |
| **Design and content** |  |
| Development process | Information about the development process of the content of the ECA |
| Development phase | The development phase of the intervention the article describes  - Development: The intervention is still subject to changes, and measures are related to usability, satisfaction, and feasibility. Measures do not yet include thorough evaluation based on relevant clinical outcomes.  - Piloting: The intervention is near completion, and relevant behavioral outcomes are considered in the evaluation. Usability, satisfaction, and feasibility outcomes can go hand-in-hand with behavioral outcomes. Evidence is not yet significant enough to give enough confidence to apply it in practice.  - Evaluation: The evaluation revolves primarily around the intervention’s effect on behavioral outcomes. Sample sizes are typically larger, and methodology is more rigorous. These interventions have been evaluated to the extent that their practical application could be considered.  - Implementation: The intervention has already gone through the evaluation phase, and has been used in practice for some time. |
| Name ECA | The name which is given to the specific ECA |
| Name intervention | The name which is given to the intervention |
| Design | Information about the design of the appearance of the ECA |
| Design process | Information about the design process of the appearance of the ECA |
| Image | An image of the ECA |
|  |  |
|  |  |
| **Support** |  |
| Aim of support | The aim of the intervention: type of lifestyle behavior the agent is targeting |
| Supported services | Other services used to support the intervention |
| Behavior change   technique | All behavior change techniques used |
| Theory | Theories or principles used to change behavior |
| Content | Information about the content the ECA offers |
|  |  |
|  |  |
| **Implementation** |  |
| Implementation | Description of the activities undertaken for the implementation of the intervention in practice |
|  |  |
|  |  |
| **Formative evaluation** |  |
| Outcome measure | The method used to collect the data: the outcome type measured  - Behavior: Whether or not the study assesses user behavior  - Knowledge: Whether or not users acquired targeted knowledge by using the intervention  - Motivation: When users report on their motivation to change  - Usability: Whether or not users have trouble using the intervention  - Usage: How often and how the intervention is used  - User satisfactions: Whether or not users respond positively to the intervention |
| Conclusion authors | The main conclusion of the study, as described in the abstract |
| Conclusion reviewers | The main conclusion of the study, by the reviewers |
