# Supplementary material for: Developing Embodied Conversational Agents for Coaching People in a Healthy Lifestyle: Scoping Review
Source: J Med Internet Res. 2020 Feb 5;22(2):e14058. doi: 10.2196/14058 (PMC7055763; doi:10.2196/14058)
Supplement: Multimedia Appendix 3 [file jmir_v22i2e14058_app3.docx]

## Multimedia appendix 3. Overview of studies

**Table 1.** Description of study design, development phase, behaviour change techniques, theory or principle, outcome variable, outcomes measures and outcome.

| Reference | Study design | Development phase | BCT | Theory | Outcome variable | Outcome measure | Outcome ^a^ |
| --- | --- | --- | --- | --- | --- | --- | --- |
| [17] | Three-armed RCT | Piloting | ‘Goal setting (behavior)’, ‘information about health consequences’, ‘instruction on how to perform the behavior’, ‘review behavior goals’, ‘problem solving’,  ‘self-monitoring of behavior’ and ‘social reward’ | Behavioral therapy, Cognitive Behavioral Therapy, Social Learning Theory and Transtheoretical Model | Behavior | Pedometer and self-report^b^ | - |
|  |  |  |  |  | Usage | Logfiles | + |
|  |  |  |  |  | User satisfaction | Interviewa and questionnaire | n.a. |
| [21] | Three-armed RCT | Piloting | Not reported | Stage models of relationships | Behavior | Pedometer and self-report^b^ | - |
|  |  |  |  |  | Usage | Logfiles | + |
|  |  |  |  |  | User satisfaction | Questionnaire | n.a. |
| [3] | Two-armed RCT | Piloting | Not reported | Models of human relationships | Behavior | Pedometer | - |
|  |  |  |  |  | Usability | Interview^b^ and questionnaire | n.a. |
|  |  |  |  |  | Usage | Logfiles | n.a. |
|  |  |  |  |  | User satisfaction | Interview^b^ and questionnaire | n.a. |
|  |  |  |  |  | Other: Well-being | Questionnaire | - |
|  |  |  |  |  | Other: Loneliness | Questionnaire | - |
| [20] | Two-armed RCT | Evaluation | ‘Credible source’, ‘discrepancy between current behavior and goal’, ‘feedback on behavior’, ‘goal setting (behavior)’, ‘social reward’, ‘information about health consequences’, ‘self-monitoring of behavior’ and ‘social support (unspecified)’ | Motivational Interviewing | Behavior | Self-report | - |
|  |  |  |  |  | Knowledge | Questionnaire | - |
|  |  |  |  |  | Motivation | Questionnaire | + |
|  |  |  |  |  | Usability | Questionnaire | + (*d*=0.38) |
|  |  |  |  |  | Usage | Logfiles | + (*d*=0.38) |
|  |  |  |  |  | Other: BMI | Calibrated scale | + |
| [4] | Two-armed RCT | Piloting | ‘Feedback on behavior’ and ‘goal setting (behavior)’ | Not reported | Usage | Logfiles | n.a. |
|  |  |  |  |  | User satisfaction | Questionnaire | n.a. |
| [22] | Two-armed RCT | Development | Not reported | Not reported | Behavior | Pedometer | n.a. |
|  |  |  |  |  | Usage | Logfiles | n.a. |
|  |  |  |  |  | User satisfaction | Questionnaire | n.a. |
|  |  |  |  |  | Other: dishonest | Questionnaire | n.a. |
| [23] | Two-armed RCT | Evaluation | ‘Commitment’, ‘goal setting (behavior)’, ‘information about health consequences, ‘non-specific reward’, ‘problem solving’,  ‘self-monitoring of outcome of behavior’, ‘social reward’ and  ‘social support (unspecified)’ | Behavioral therapy, Social Cognitive Theory | Behavior | Pedometer and questionnaire^b^ | + |
|  |  |  |  |  | Usage | Logfiles | - |
|  |  |  |  |  | User satisfaction | Questionnaire | - |
|  |  |  |  |  | Other: weight | Calibrated scale and questionnaire^b^ | - |
| [24] | Pretest-posttest design without control group | Piloting | ‘Information about health consequences’ | Patient-centered care, Transtheoretical Model | Motivation | Questionnaire | n.a. |
|  |  |  |  |  | Usability | Questionnaire | n.a. |
|  |  |  |  |  | Usage | Logfiles | n.a. |
|  |  |  |  |  | User satisfaction | Questionnaire | n.a. |
| [18] | Pretest-posttest design without control group | Piloting | ‘Feedback on behavior’, ‘goal setting (behavior)’,  ‘information about health consequences’,  ‘non-specific reward’,  ‘problem solving’, ‘self-monitoring of outcome of behavior’ and ‘social support (practical)’ | Behavioral change theory, Cognitive Behavioral Therapy | Behavior | Walking test | n.a. |
|  |  |  |  |  | Usage | Logfiles | n.a. |
|  |  |  |  |  | User satisfaction | Questionnaire | n.a. |
|  |  |  |  |  | Other: safety | Phone calls | n.a. |
| [19] | Four-armed RCT | Piloting | ‘Feedback on behavior’, ‘goal setting (behavior)’, ‘information about health consequences’, ‘monitoring of behavior’ and ‘social reward’ | Social Cognitive Theory, Transtheoretical Model | Behavior | Questionnaire | +( *d*=1.2) |
|  |  |  |  |  | Motivation | Questionnaire | + (*d*=0.6-1.2) |
|  |  |  |  |  | Usage | Logfiles | n.a. |
|  |  |  |  |  | Usability | Not reported | n.a. |
|  |  |  |  |  | User satisfaction | Questionnaire | n.a. |
|  |  |  |  |  | Other: safety | Not reported | n.a. |
| [25] | Four-armed RCT | Piloting | ‘Action planning’, ‘goal setting (behavior)’,  ‘information about health consequences’, ‘problem solving’, ‘review behavior goals’ and ‘reviewing progress’ | Motivational Interviewing Social Cognitive Theory and Transtheoretical Model | Behavior | Pedometer and questionnaire^b^ | n.r. |
|  |  |  |  |  | Usability | Questionnaire | n.a. |
|  |  |  |  |  | User satisfaction | Interview^b^ and questionnaire | n.a. |
|  |  |  |  |  | Other: weight | Calibrated scale | - |
| [26] | Two-armed pseudo-RCT | Piloting | ‘Action planning’, ‘demonstration of the behavior’, ‘information about health consequences’, ‘instruction on how to perform the behavior’,  ‘social reward, problem solving’ and ‘verbal persuasion about capability’ | Mindfulness Based Stress Reduction | Knowledge | Questionnaire | n.r. |
|  |  |  |  |  | Motivation | Questionnaire | n.r. |
|  |  |  |  |  | Usage | Logfiles | + (*d*=>1.2) |
|  |  |  |  |  | User satisfaction | Questionnaire | n.r. |
| [27] | Two-armed RCT | Evaluation | ‘Goal setting (behavior)’, ‘problem solving’, ‘reviewing progress’, ‘self-monitoring of outcome of behavior’, ‘social reward’ and ‘social support (practical)’ | Not reported | Behavior | Pedometer | + |
|  |  |  |  |  | Usage | Logfiles | n.a. |
|  |  |  |  |  | User satisfaction | Questionnaire | n.a. |
|  |  |  |  |  | Other: adverse events | Not reported | n.a. |
| [32] | Three-armed RCT | Evaluation | ‘Goal setting (behavior)’, ‘problem solving, information about health consequences’ and ‘verbal persuasion about capability’ | Motivational Interviewing | Behavior | Questionnaire | + |
|  |  |  |  |  | User satisfaction | Questionnaire | n.a. |
| [28] | Two-armed RCT | Piloting | ’Action planning’, ‘goal setting (behavior), ‘information about health consequences’, problem solving and ‘social support (practical) | Motivational Interviewing and Transtheoretical Model | Behavior | Questionnaire | + |
|  |  |  |  |  | Usage | Logfiles | n.a. |
|  |  |  |  |  | User satisfaction | Questionnaire | n.a. |
| [33] | Three-armed RCT | Piloting | ‘Feedback on behavior’, ‘self-monitoring of behavior’ and ‘social support (practical)’ | Transtheoretical Model | Behavior | Activity monitor | - |
|  |  |  |  |  | Usability | Questionnaire | n.a. |
|  |  |  |  |  | Usage | Logfiles | n.a. |
|  |  |  |  |  | User satisfaction | Interview^b^ and questionnaire | - |
| [29] | Cluster RCT (report on design) | Evaluation | ‘Feedback on behavior’, ‘goal setting (behavior)’, ‘non-specific reward’, ‘self-monitoring of behavior’, ‘social reward’ and ‘social support (unspecified)’ | Social Cognitive theory, Transtheoretical Model |  |  |  |
| [30] | Two-armed RCT | Piloting | ‘Action planning’, ‘demonstration of the behavior’, ‘goal-setting (behavior)’, ‘information about health consequences’, ‘instruction on how to perform the behavior’, ‘problem solving’ and ‘social support (practical)’ | Motivational Interviewing, self-management and shared decision-making | Behavior | Interview | - |
|  |  |  |  |  | Knowledge | Interview | - |
|  |  |  |  |  | Usage | Logfiles | n.a. |
|  |  |  |  |  | User satisfaction | Interview^b^ and questionnaire | - |
|  |  |  |  |  | Other: feasibility | Recruitment | n.a. |
|  |  |  |  |  | Other: stress management techniques | Questionnaire | - |
| [5] | Pretest-posttest design without control group | Piloting | ‘Feedback on behavior’, ‘goal setting (behavior)’, ‘information about health consequences’, prompts/cues’, ‘punishment’, ‘self-monitoring of behavior’, ‘self-monitoring of outcome of behavior’, social reward, ‘social support (practical)’ and ‘social support (unspecified)’ | Goal-setting, Persuasive System Design model and Self-determination theory | Motivation | Questionnaire | n.a. |
|  |  |  |  |  | Usage | Interview | n.a. |
|  |  |  |  |  | User satisfaction | Focus group^b^ and interview | n.a. |
|  |  |  |  |  | Other: stress | Questionnaire | n.a. |
| [31] | Pretest-posttest design with control group | Evaluation | ‘Feedback on behavior’, ‘goal setting (behavior)’, ‘prompts/cues’, ‘problem solving’, ‘self-monitoring of behavior, ‘social reward’, ‘social support (emotional)’, ‘social support (practical)’ and ‘verbal persuasion about capability’ | Transtheoretical Model | User satisfaction | Interview | n.a. |
|  |  |  |  |  | Other: reason for participation | Interview | n.a. |

^a^Outcome indicates whether there was a significant positive difference between intervention group with and control group without an embodied conversational agent.

^b^This outcome measure was seen as less objective, and was therefore disregarded.

^c^N.a.: Not applicable.

^d^N.r.: Not reported.
